# Supplementary material for: The impact of self-report inaccuracy in the UK Biobank and its interplay with selective participation
Source: Nat Hum Behav. 2024 Dec 18;9(3):584–94. doi: 10.1038/s41562-024-02061-w (PMC11936832; doi:10.1038/s41562-024-02061-w)
Supplement: Supplementary file 1 — Supplementary Methods and Figs. 1–7. [file 41562_2024_2061_MOESM1_ESM.pdf]

nature human behaviour

Supplementary information

https://doi.org/10.1038/s41562-024-02061-w

The impact of self-report inaccuracy in the UK Biobank and its interplay with selective participation

In the format provided by the authors and unedited

Table of Contents

sMethods **2**

UK Biobank probability weights *2*

sFigure 1. Impact of error in the phenotype on genotype-phenotype associations **3**

sFigure 2. Box and scatter plots for self-report measures containing outlier values **4**

sFigure 3. Concordance between objective versus subjective measures **5**

sFigure 4. Effects of age and follow up duration on residual scores (RES_i) **6**

sFigure 5. Sex differences in residual scores **7**

sFigure 6. Box and scatter plots for self-report measures included in Principal Component Analysis **8**

sFigure 7. Phenotypic variance explained by genetic variants reaching genome-wide significance **9**

sReferences **9**

sMethods

UK Biobank probability weights

To explore patterns of covariation between reporting error other participatory behaviours that are known to bias genome-wide estimates, we also included 'UKBB participation probabilities' in the analytical pipeline. The probability weights included in this work were obtained from a previous study focusing on participation bias correction in the UK Biobank[1]. A number of robustness checks were implemented to assess the performance of the probability weights, including (1) validation work using the Health Survey England[2] and the UK Census Microdata[3] (n=22,646 and n= 895,649, respectively) and (2) negative control analyses via weighted genome-wide association analysis on sex. With respect to (1), re-weighing UK Biobank participant recovered phenotypic associations as estimated in two representative UK sample. To illustrate, in the (unweighted) UK Biobank sample, there was no phenotypic correlation between age and overall health (r= -0.01), while the observed correlations are in the expected direction in the UK Census Microdata (r= -0.17) and Health Survey England (r= -0.13). Applying the UKBB probability weights recovered these observed correlations (r= -0.13). In addition (2), previous research[4] has shown that autosomal heritability linked to biological sex could result from sex-differential participation. Comparing the (uncorrected) genome-wide summary statistic results on sex (>2,400,000 participants) to those obtained from weighted genome-wide analyses showed that the application probability weighting reduced artifactual sex-heritability and SNP effects, providing evidence of diminished (sex-associated) participation bias when increasing sample representativeness.

sFigure 1. Impact of error in the phenotype on genotype-phenotype associations

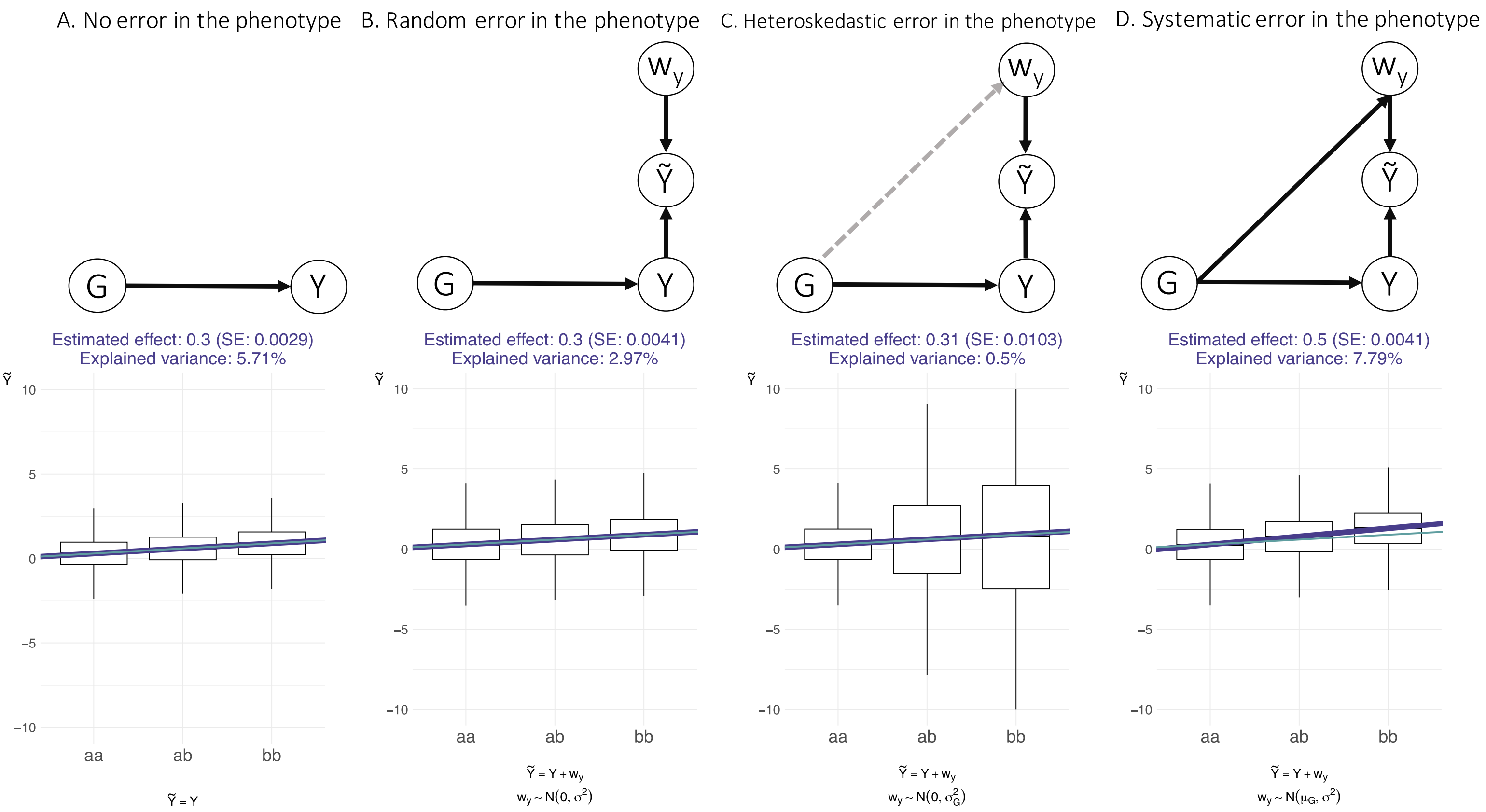

Illustration of possible measurement error mechanism when testing the effect of a single genetic variants (G, assumed to be measured without error) on a continuous phenotype measured without error (Y) or with error ($\tilde{Y}$). Data was simulated for a sample of n=180,000. $\tilde{Y}$, the observed phenotype, is a function of the true value of the phenotype (Y) and the error in the phenotype (w_y), i.e., $\tilde{Y} = Y + w_y$. Biallelic SNP genotypes (aa, ab, bb) are presented on the X-axes. Random error in the phenotype (illustrated in **panel B**) occurs when w_y is unrelated to G and Y, here simulated by adding a random normal variable with a mean of zero and a standard deviation of one (i.e., constant or homoskedastic error variance) to the model. Random error in the phenotype does not induce bias in SNP effects, but increases the standard errors and reduces the variance explained by the genotype. Heteroskedastic error in the phenotype (illustrated in **panel C**) occurs when the variance in w_y depends on G (e.g., where bb-carriers show larger random errors than aa-carriers, i.e., error that is not constant across G). Heteroskedastic error does not induce bias in the SNP effects, but leads to incorrect standard errors. Systematic error (i.e., error that is not random and where the direction of the error is different across G, illustrated in **panel D**) occurs if the mean in w_y depends on G (μ_{aa}=0, μ_{ab}=0.2, μ_{bb}=0.4). Systematic error results in biased SNP estimates.

sFigure 2. Box and scatter plots for self-report measures containing outlier values

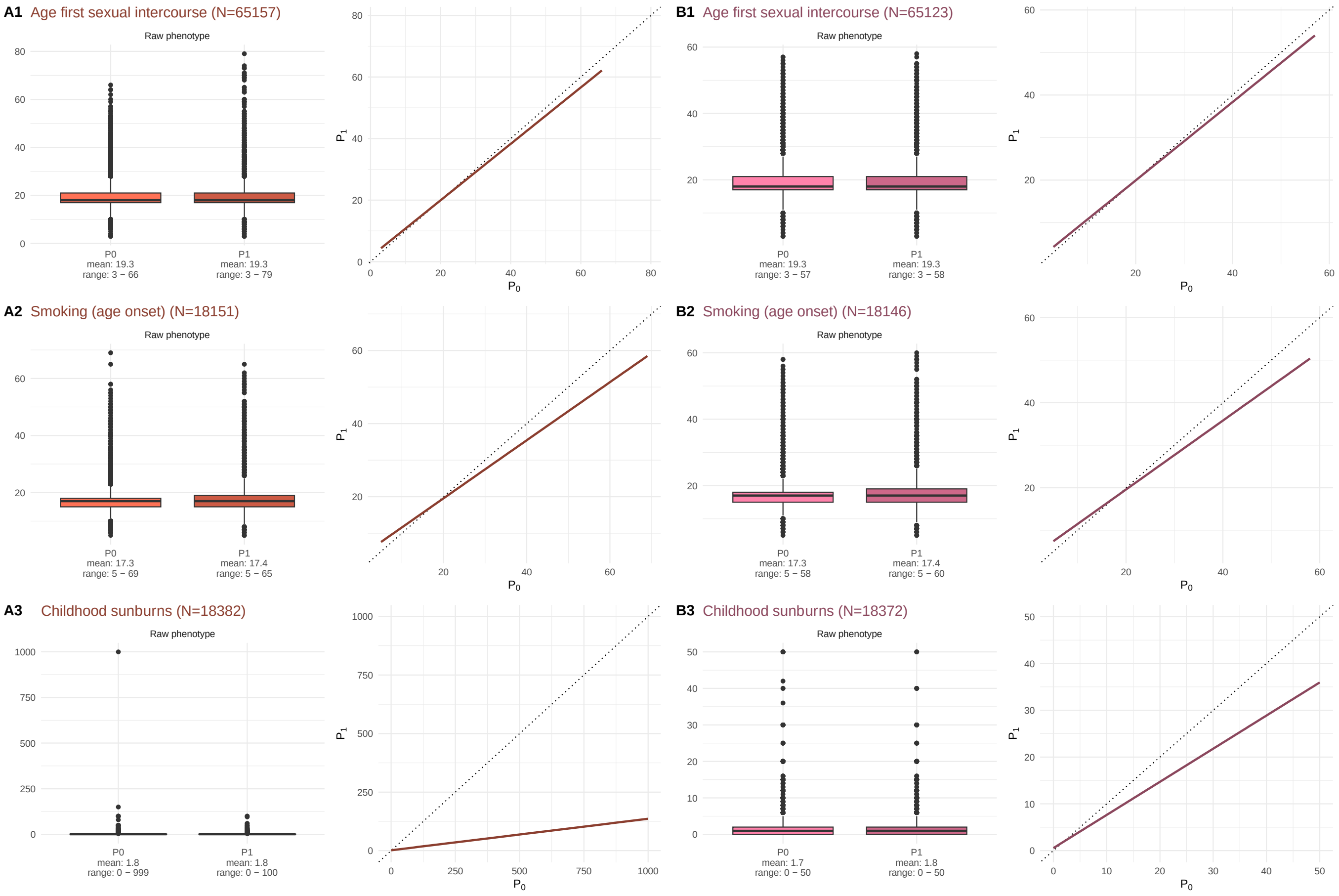

Panel A1-A3 display the box and scatter plots of the self-report measure assessed at baseline (P_0) and follow-up (P_1) prior to outlier removal. Mean, minimum and maximum values for each phenotype are provided on the x-axis. Panel B1-B3 display the same information after the removal of outlier values.

sFigure 3. Concordance between objective versus subjective measures

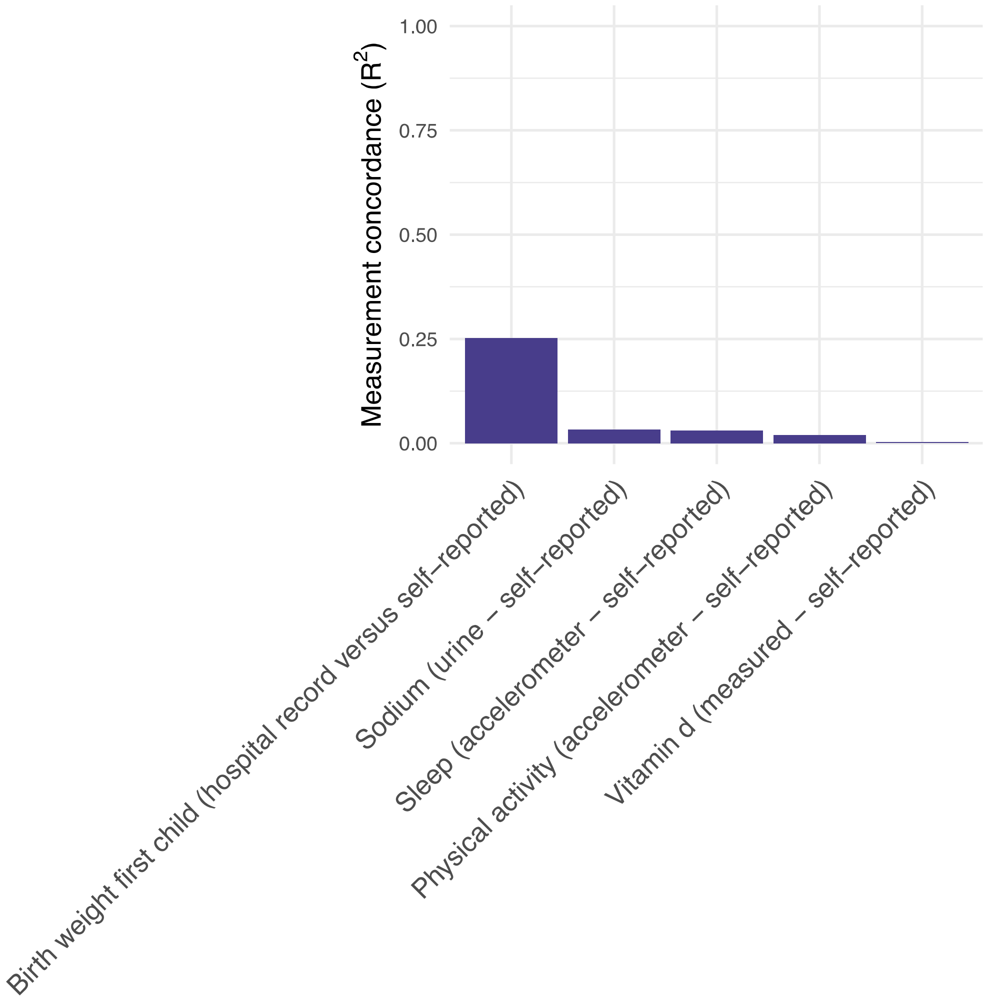

Measurement concordance (R^2) comparing objective and subjective measures for five UKBB phenotypes. R^2=Variance explained by models regressing objectively ascertained phenotype (e.g., accelerometer derived sleep duration) onto the subjectively ascertained phenotype (e.g., self-reported sleep duration), while controlling for follow up time. The following UKBB variables were used: Birth weight of the first child, including self-reported (ID 2744, 'what was the birth weight of your first child in pounds?') and hospital recorded (ID 41284, the birth weight of the first baby born to the participant in their hospital inpatient records); Physical activity (as done in previous studies[5]), including self-reported (ID 22040, the total Metabolic Equivalent Task minutes per week) and accelerometer derived (ID 90019-90025, used to derive an acceleration average. Individuals with poor wear time (ID 90015) were excluded); sleep duration, including self-reported (ID 1160, 'about how many hours sleep do you get in every 24 hours?') and accelerometer derived (ID 90027-90050, used to derive the average number of hours of sleep per 24 hours, applying a cut-off of $\leq$10 milligravity to indicate sleep. Individuals with poor wear time (ID 90015) were excluded); sodium intake, including self-reported (ID 26052, sodium intake obtained from the 24-hour dietary recall questionnaire) and measured (ID 30530, sodium measured in urine); vitamin D intake, including self-reported (ID 100021, vitamin D obtained from the 24-hour dietary recall questionnaire) and measured (ID 30890, vitamin D obtained from blood measures).

sFigure 4. Effects of age and follow up duration on residual scores (RES_i)

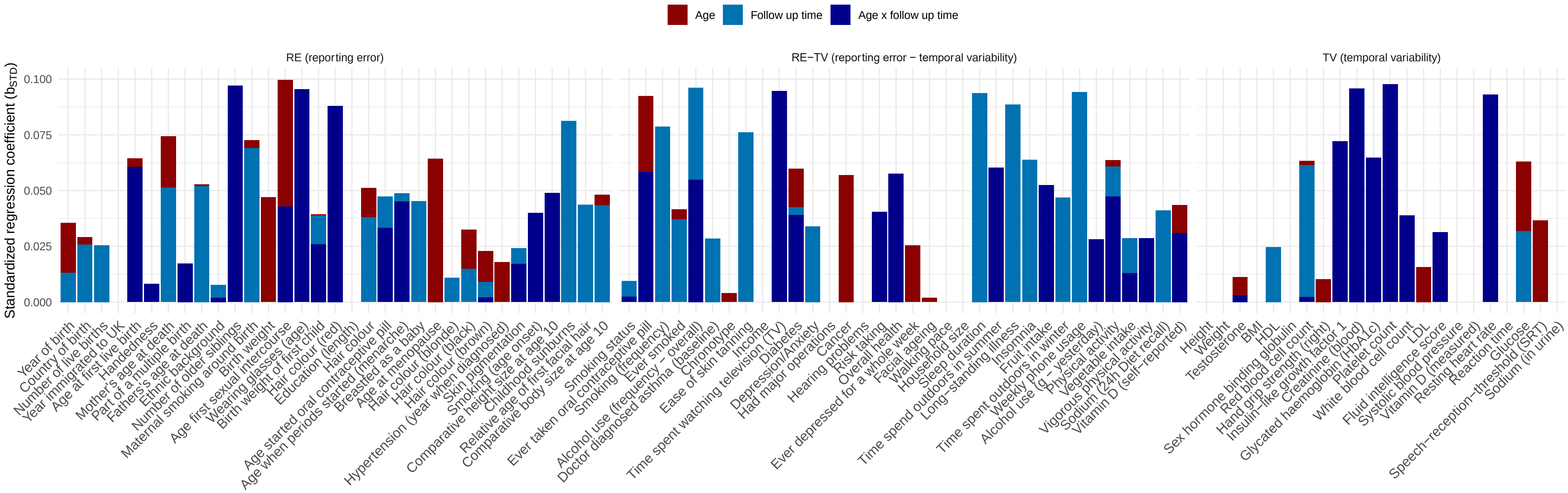

Standardized regression coefficients of age and follow up duration on the raw residual scores. RES_i are derived from a model regressing the phenotype measured at time point 2 (P_{T2}, e.g., birth weight reported at follow up) onto the phenotype assessed at time point 1 (P_{T1}, e.g., self-reported birth weight assessed at baseline).

sFigure 5. Sex differences in residual scores

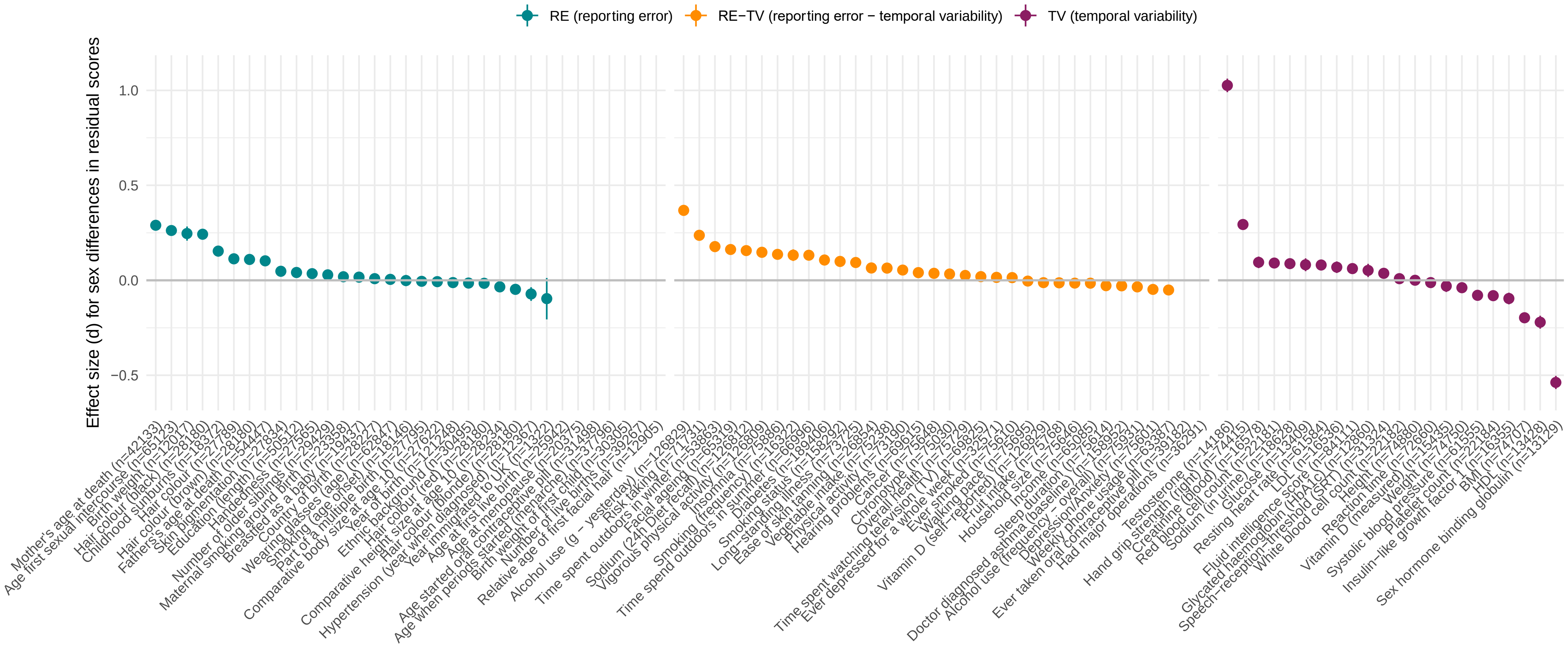

Estimates of Cohen's *d* quantifying sex differences in residual scores. The residual scores are derived from a model regressing the phenotype measured at time point 2 (P_{T2}, e.g., birth weight reported at follow up) onto the phenotype assessed at time point 1 (P_{T1}, e.g., self-reported birth weight assessed at baseline), while controlling for follow up time ($time_{T2\text{-}T1}$). Traits with missing estimates of Cohen's *d* index those that are assessed in either males (e.g., age of first facial hair) or females (e.g., age at menopause) only. All error bars shown in the figure represent the 95% confidence intervals.

sFigure 6. Box and scatter plots for self-report measures included in Principal Component Analysis

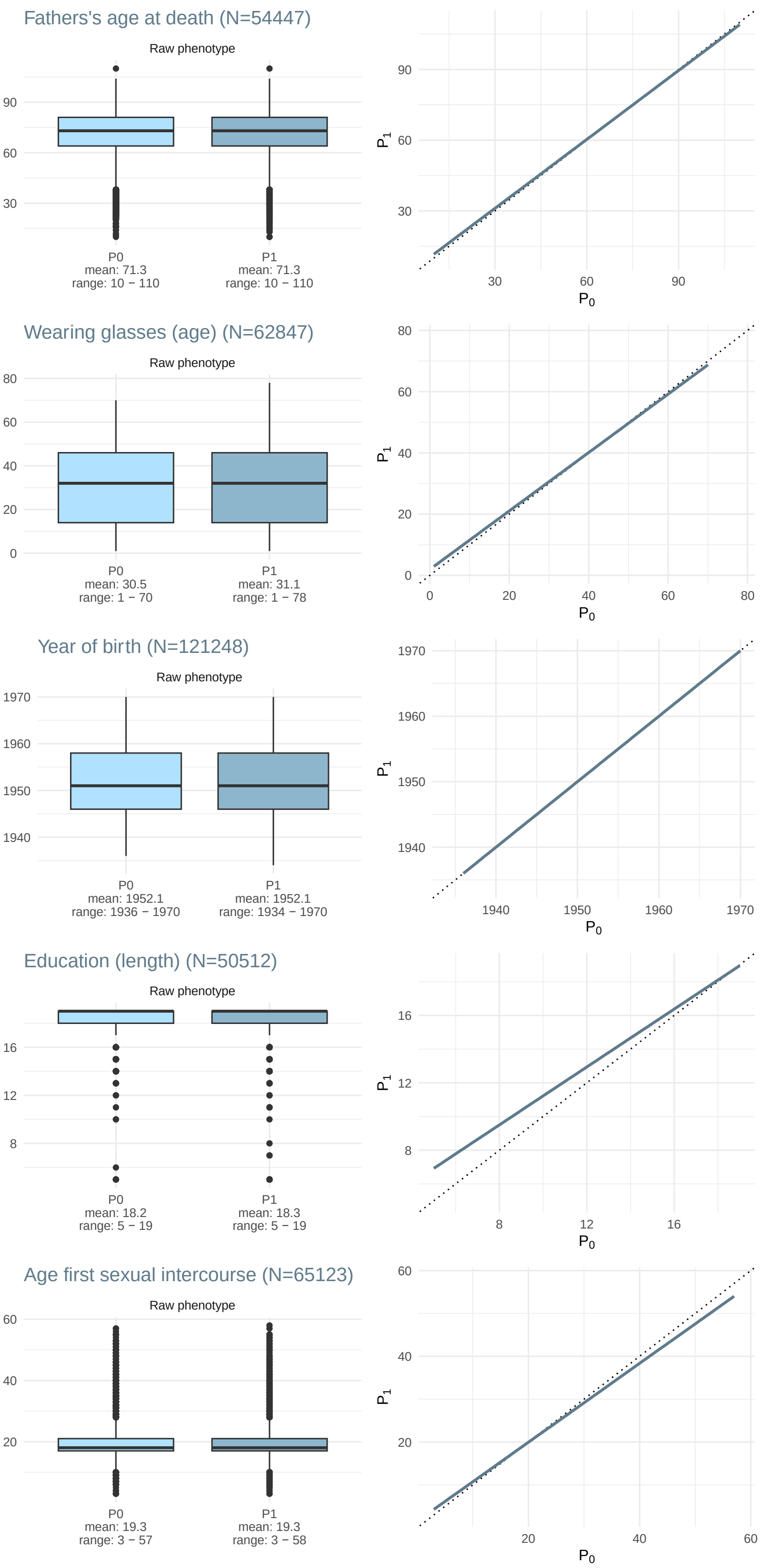

The figure displays the box and scatter plots of the self-report measures used in Principal Component Analysis, including the phenotype assessed at baseline (P_0) and follow-up (P_1). Mean, minimum and maximum values for each phenotype are provided on the x-axis.

sFigure 7. Phenotypic variance explained by genetic variants reaching genome-wide significance

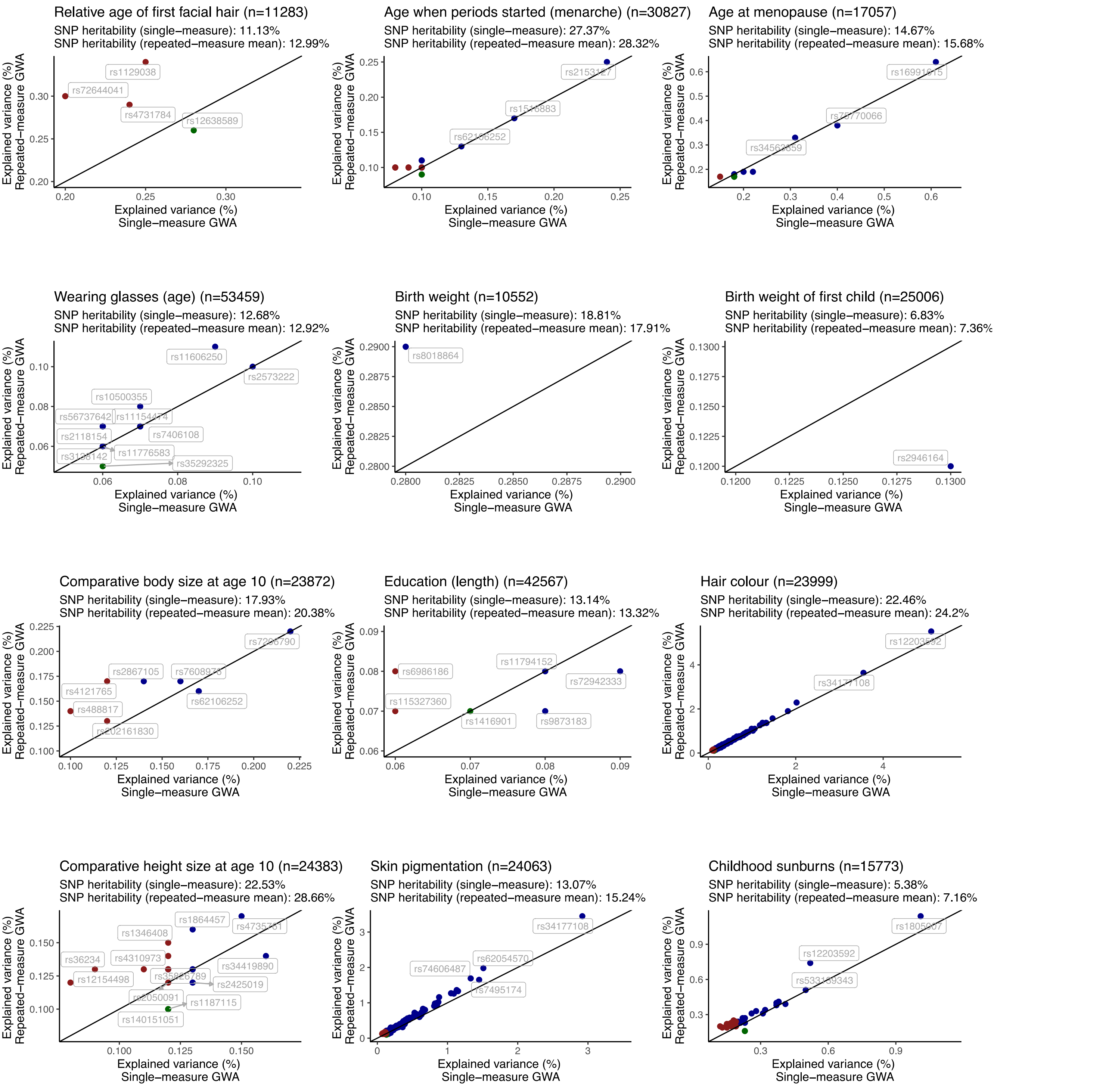

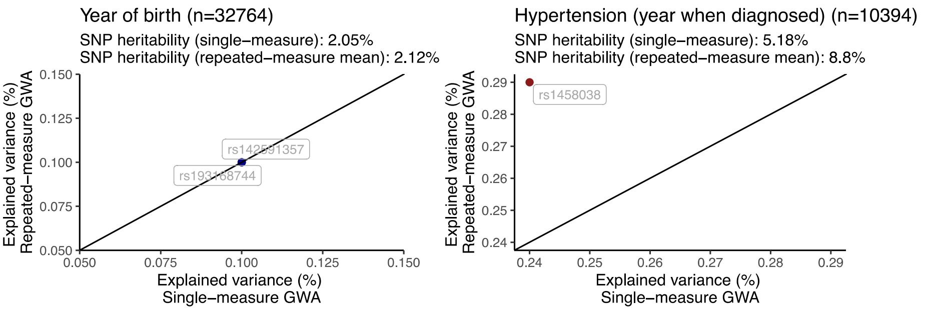

Explained variance (β_{STD}^2) per SNP reaching genome-wide significance in error-corrected GWA analyses (y-axis, phenotype obtained using means across multiple measurement occasions) or error-uncorrected GWA analyses (x-axis, phenotype obtained from a single baseline measure). The colour scheme highlights in which GWA the genetic variant was identified, including error-corrected GWA (in red), error-uncorrected GWA (in green) or in both (blue).

sReferences

1. Schoeler T, Speed D, Porcu E, Pirastu N, Pingault J-B, Kutalik Z. Participation bias in the UK Biobank distorts genetic associations and downstream analyses. *Nat Hum Behav*. Published online April 27, 2023. doi:10.1038/s41562-023-01579-9
2. Mindell J, Biddulph JP, Hirani V, et al. Cohort Profile: The Health Survey for England. *Int J Epidemiol*. 2012;41(6):1585-1593. doi:10.1093/ije/dyr199
3. 2011 Census Microdata. Office for National Statistics. Published 2011. https://www.ons.gov.uk/census/2011census/2011censusdata/censusmicrodata
4. Pirastu N, Cordioli M, Nandakumar P, et al. Genetic analyses identify widespread sex-differential participation bias. *Nat Genet*. 2021;53(5):663-671. doi:10.1038/s41588-021-00846-7
5. Guo W, Key TJ, Reeves GK. Accelerometer compared with questionnaire measures of physical activity in relation to body size and composition: a large cross-sectional analysis of UK Biobank. *BMJ Open*. 2019;9(1):e024206. doi:10.1136/bmjopen-2018-024206
